# Volume Completion Between Contour Fragments at Discrete Depths

**DOI:** 10.1177/2041669517747001

**Published:** 2017-12-21

**Authors:** Peter Ulric Tse

**Affiliations:** Department of Psychological and Brain Sciences, 3728 Dartmouth College, Hanover, USA

**Keywords:** 3D perception, binocular vision, contours/surfaces, grouping, perceptual organization, shape, surfaces/materials

## Abstract

Building on the modal and amodal completion work of Kanizsa, Carman and Welch showed that binocular stereo viewing of two disparate images can give rise to a percept of 3D curved, nonclosed illusory contours and surfaces. Here, it is shown that binocular presentation can also give rise to the percept of closed curved surfaces or volumes that appear to vary smoothly across discrete depths in binocularly fused images, although in fact only two binocular disparities are discretely defined between corresponding contour elements of the inducing elements. Surfaces are filled in from one depth layer’s visible contours to another layer’s visible contours within virtual contours that are interpolated on the basis of good contour continuation between the visible portions of contour. These single depth contour segments are taken not to arise from surface edges, as in Kanizsa’s or Carman and Welch’s examples, but from segments of “rim” where the line of sight just grazes a surface that continues behind and beyond the rim smoothly. When there are two or more surface-propagating contour segments, the propagated surfaces can continue away from the inferred rim, merge, and then close behind the self-occluding visible surface into an everywhere differentiable closed surface or volume. Illusory surfaces can possess a depth and perceived surface curvature that is consistent with all visible contour segments, despite the absence of local disparity cues at interpolated 3D surface locations far from any visible contour. These demonstrations cannot be easily explained by existing models of visual processing. They place constraints on the surface and volume generation processes that construct our 3D world under normal viewing conditions.

## Introduction

In the late 1990s, several researchers began providing evidence (Albert & Tse, 2000; Tse, 1998; Tse & Albert, 1998; Tse 1999a, 1999b, 2002; Van Lier, 1999; Van Lier & Wagemans, 1999) that amodal and modal completion take place at a 3D object or volumetric level of representation, rather than at the level of image contours or the level of visible surfaces. By “volume” is meant a 3D interpolated closed surface, including the invisible but sensed backside of a visible surface (Ekroll, Sayim, & Wagemans, 2013; Ekroll, Sayim, Van Der Hallen, & Wagemans, 2016), and including, as well, the interpolated spatial inside that it encloses (Tse, 1999a, 1999b). Here, I build on this tradition, by introducing a novel class of stereoscopic volume completion illusions that places strong constraints on the surface and volume generation processes that construct our 3D world under normal viewing conditions. These demonstrations raise issues that cannot be easily explained by existing models of visual processing.

This work builds on and extends the insights provided by the drawing of a “Kanizsa triangle” (Kanizsa, 1955), where a flat illusory triangle appears to float above black disks that are occluded by that triangle. The key point made by Kanizsa’s demonstration is that surfaces are interpolated on the basis of both contour cues and cues about visual occlusion provided by black “pacmen,” which were in fact the only elements actually present in his drawing. The types of examples of amodal and modal completion given in Kanizsa (1995) or Carman and Welch (1992) involve open surfaces that complete modally in front of pacmen-like inducers. The visible and illusorily completed contours of these unclosed Kanizsa-style figures correspond to an edge in the world where the surface is taken to just end, like the edge of a piece of paper. The new demonstrations shown here, in contrast, involve visible and illusorily completed contours that are generally inferred to arise not from edges, but instead from portions of the “rim,” where the line of sight is taken to just graze, tangentially, a smooth or differentiable surface. Because illusory surfaces are taken to continue before, behind, and beyond the visible or illusory contour arising from the rim of the modally completing surface, they can close into a volume that encloses space. Thus, curved 3D surfaces are interpolated by the visual system to vary smoothly across depths in binocularly fused images, even when only two (or more) discrete binocular disparities are defined between corresponding elements of the inducing image contours. These illusory surfaces are generated in the 3D space inferred to lie between the two (or more) disparity-defined depths, and only arise in uniform regions where there are no disparity cues that could define depth upon binocular fusion. Surfaces are filled in from one depth layer’s visible contour fragments to another layer’s visible contour fragments within virtual contours that are themselves interpolated on the basis of good contour continuation (i.e., the extent to which contours connect modally or amodally as a function of the degree of interpolated coalignment in space; Tse, 1998; Tse, 1999a, 1999b). Depending on global contour geometry, curved surfaces may be interpolated to be open or closed, in which case they form volumes. Such interpolated 3D surfaces may pass through visible contours along the line of sight, or at some other angle. The interpolated surface solution is influenced by nonlocal cues: When there are two or more surface-propagating contour segments, they can merge and possess a depth and perceived surface curvature that is consistent with all visible contour segments, despite the absence of local disparity cues in regions far from any inducing contours. Indeed, because surfaces are assumed to close smoothly, there are cases where the interpolated curved closed surfaces appear to lie closer or farther than the nearest or farthest depth respectively implied by binocular disparity cues at visible contours.

## Historical Background

**Figure 1.**
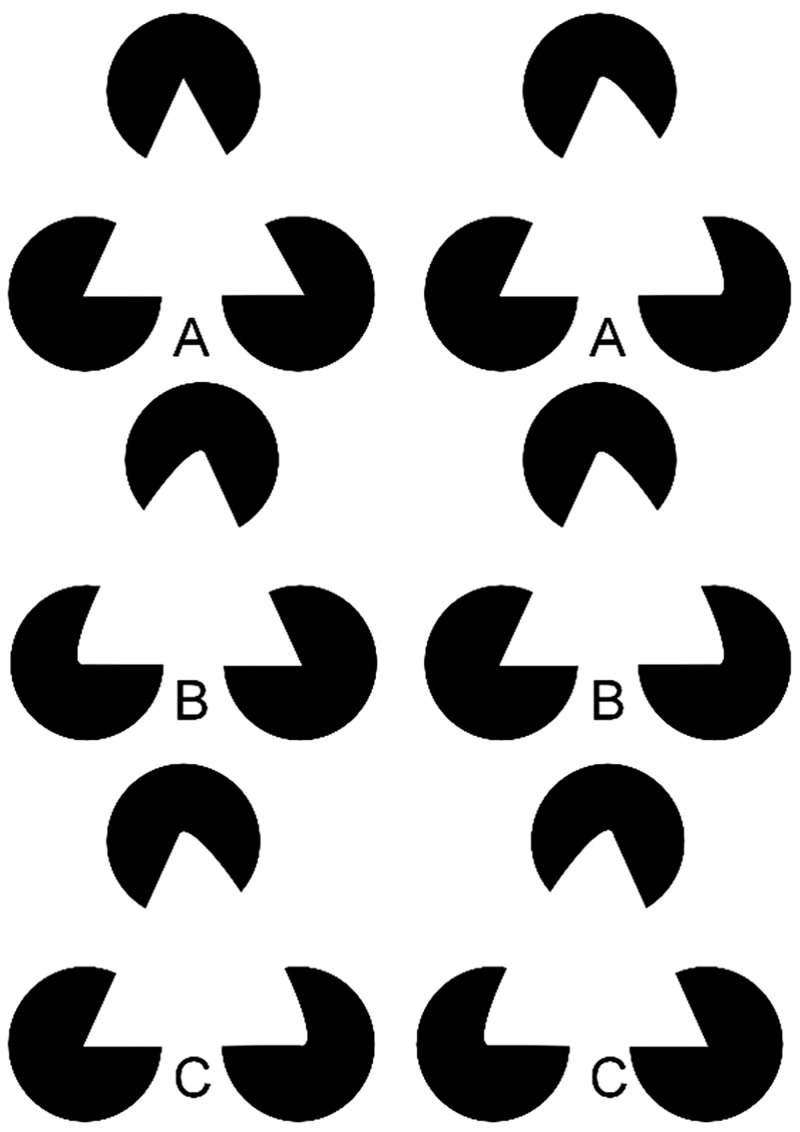
Cross-fusion of the horizontal pairs of images permits the perception of 3D curved surfaces. (For an uncrossed disparity version, see Figure S1.) In order to cross-fuse two horizontally displaced images, cross the eyes until four rather than two images are seen, and then alter the degree to which the eyes are crossed until just three images are visible, the middle of which will appear in 3D. For example, in (A) one could cross-fuse either the triangle itself, or the letter “A” at the bottom of (A). Doing so will result in a percept in (A) of a 3D cone opening to the right; in (B), a convex curved version of a Kanizsa triangle; and in (C), a concave curved Kanizsa triangle viewed through apparent circular “windows.”

The perception of 3D surfaces can arise even in the absence of binocular disparity cues. Such surfaces are constructed by the observer’s own visual system when viewing the Kanizsa triangle drawing, for example, which inspired the binocular cases shown in Figure 1. These drawings follow Carman and Welch (1992), who were the first to show stereoscopically defined curved surfaces. The Kanizsa triangle or these 3D curved versions make apparent that a problem arises for visual processing of object shape, completion, and depth ordering, when only some contours of an object are visible in the image, whether because of camouflage or occlusion, including self-occlusion. Kanizsa’s triangle demonstrated that the visual system solves this problem constructively by filling in missing edges and surfaces of the occluding triangle in what is known as “modal completion,” and connects surfaces behind occluders, in what is known as “amodal completion,” as occurs for the black “pacmen,” which appear to be solid disks behind the modally completed occluding triangle. The visual system thus interpolates information that is not present in the image and our conscious visual experience is not of information detected by early visual processes, but is rather of information constructed on the basis of earlier detected information.

Monocular cases of 3D surface completion, modal and amodal completion, as found in the Kanizsa triangle, can be made even more compelling by placing surfaces in front of or behind one another using stereopsis. The perception of 3D structure on the basis of stereopsis was explained by Charles Wheatstone (1838): “…  the mind perceives an object of three dimensions by means of the two dissimilar pictures projected by it on the two retinae …” (pp. 372–373). Because each eye is displaced horizontally from the other, the two eyes’ views differ. The degree of horizontal disparity between corresponding points of the two images will vary with the depth of the objects that project those images. This discovery led to the stereoscope craze of the late 1800s, followed more recently by popular fads involving viewmasters, magic eye books, and 3D films.

Wheatstone did not consider the generation of curved surfaces under stereopsis. Some examples of such curved surfaces, following the work of Carman and Welch (1992), are shown in Figure 1 using crossed disparities (For an uncrossed disparity version, see Figure S1). In the top row (A) of Figure 1, if the eyes are crossed until three rather than two or four illusory triangles (or letters) are seen, a 3D cone, or perhaps semicone,^1^ opening to the right appears to float above the pacmen, and the illusory contours of the constructed shape are apparent. In the middle row (B), a convex curved triangle is seen, whereas in the bottom row (C), the concave curved triangle appears to be at a greater depth from the viewer than the black inducers, forcing an entirely different interpretation of the scene, namely one where there are three circular windows through which one sees an amodally completing concave triangle. Note that for each point on the contour of the triangle of one image, there is a unique matching point in the other image. There are no unmatched points between the images in the two eyes, and disparities vary in an analog manner along the contour of the inferred triangles in Figure 1.

**Figure 2.**
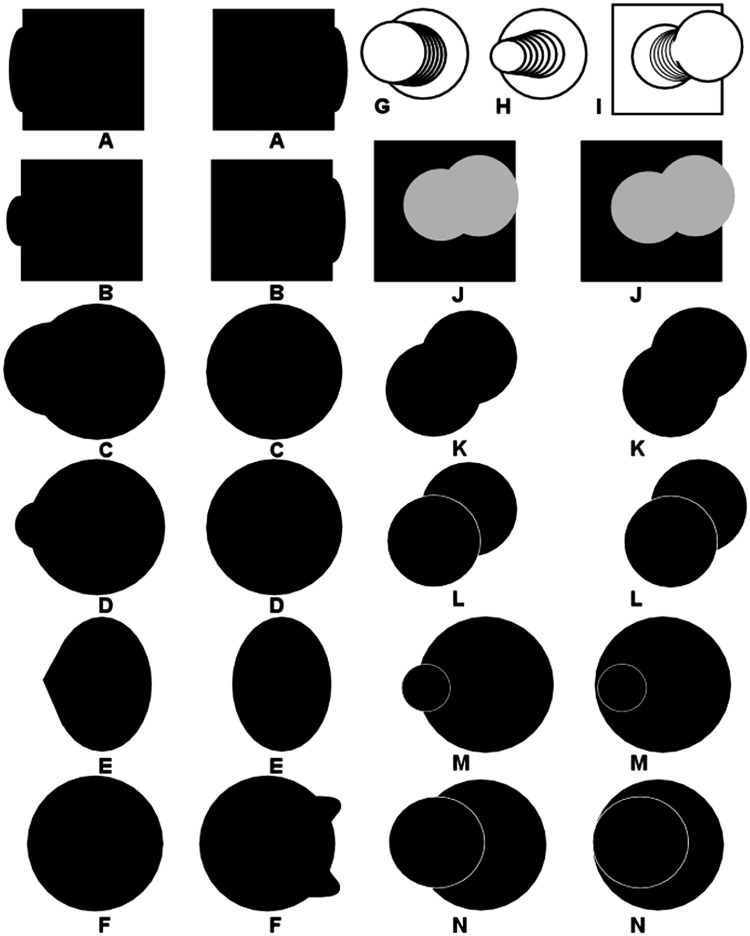
Cross-fusing the horizontal pairs of images results in the perception of (A) a 3D cylinder or half-cylinder ambiguously either floating in front of a rectangle or attached to it, (B) a 3D flask, (C) a “top hat” or “bowler hat” with the shape shown in (G), (D) a “witch’s hat” with the shape shown in (H), in (E) a “Chinese hat,” and in (F) a 3D jutting “duckbill.” Note that (A) and (B) are constructed from two squares and two vertical ellipses, whereas (C) and (D) are constructed from overlapping circles. Making the boundaries of those circles explicit, as in (M) and (N), blocks surface propagation and volume completion, and only two flat disks are perceived, one in front of the other. Cross-fusing (J) results for many viewers in the volumetric perception of the “coat-rack peg” depicted in (I), as does (K) from a different viewing angle. Both (J) and (K) are made by overlapping two disks. But making the boundaries of these disks explicit as in (L) again blocks 3D surface propagation between disparity-defined depths, and only one disk floating in front of another is perceived. For an uncrossed disparity version of this figure, see Figure S2.

Other examples of disparity-defined 3D curved surfaces exploit a phenomenon called “da Vinci stereopsis” (Cao & Grossberg, 2005; Nakayama & Shimojo, 1990; Wardle & Gillam, 2013). Leonardo da Vinci considered cases where an occluder that is entirely visible to both eyes occludes a second more distant surface. The occluded surface has regions that are only visible to one eye, and each eye can see portions of the farther surface that the other eye cannot see. The examples in Figure 2 involve what might be called “reverse da Vinci stereopsis,” where portions of the *occluding* surface or contour are only visible to one eye or the other. (For an uncrossed disparity version, see Figure S2.) Because a noncorresponding edge is only visible to one eye, there will be “missing edges” in the interior of a figure that do not give rise to a disparity signal, because a disparity signal requires corresponding features or edges that are visible to each eye. This absence of a “blocking boundary” permits an occluding surface to “propagate” toward edges potentially located at different depths. In the case of Figure 2(A) and (B), the surfaces propagate across the occluded background to form an occluding “cylinder” or “flask,” respectively. (Interestingly, if one gently rotates one’s head around the axis that passes between the bridge of the nose and the inion, the perceived volume appears to deform.) The example shown in Figure 2(C) is of particular interest because the only surface to which propagation could take place from the unmatched front layer is toward the back layer. Remarkably, most observers report being able to see a volumetric shape (i.e., a closed 3D surface) in the form of a “top hat” or “bowler hat” viewed from above. The flat top hat interpretation is drawn in Figure 2(G), as one of the dominant 3D percepts evoked. Note, however, when the corresponding edges of the occluder are made explicit so that there are no unmatched edges between the two eyes’ images, as in Figure 2(N), no surface propagation is possible between depth layers because the visible edge has explicit disparities at every location, which blocks the possibility of interpolating or propagating that surface to disparity-free regions distant from and outside the boundaries imposed by the visible disk. This forces contours to be interpreted as edge rather than rim. Thus, if partial occluding contours are placed in front, via reverse da Vinci stereopsis, the lack of bounding contours at the same perceived depth permits the disparity-free uniform regions to be interpolated as transitioning smoothly between layers. Several other examples of this are shown in the rest of Figure 2. Cross-fusing Figure 2(D) leads to the volumetric perception of a “witch’s hat” like that drawn in Figure 2(H). Note that in both of these “hat” cases, there are actually only two discrete depths defined by the disparity signals between two overlapping black circles. Again, making contours apparent, as in Figure 2(M), blocks surface propagation from occurring across depths and forces a surface edge rather than rim interpretation of visible contours. Figure 2(E), in turn, looks like a “Chinese farmer’s hat.” Cross-fusing Figure 2(F) leads to the illusory volumetric perception of a smoothly protruding “duck bill” even though only two depth disparities are defined in the image. Figure 2(J) and (K) are also constructed using just two overlapping disks. Unlike the reverse da Vinci stereopsis cases shown in Figure 2(C) and (D), there are no entirely unmatched contours in Figure 2(J) and (K). Nonetheless, there are unmatched portions of the occluding front circle’s contour. This is sufficient to allow the inferred surface to propagate away from the front layer toward the back layer, creating the impression that one is viewing a “hat-rack peg” from below in Figure 2(J) and above in Figure 2(K), with the approximate perceived shape depicted in Figure 2(I). Making the edges of the front layer of Figure 2(K) explicit, as in Figure 2(L), blocks propagation of the interpolated surface between layers, and one experiences one disk occluding another rather than a peg-like volume. All these cases involve smooth interpolation of surfaces into zero-disparity regions between just two disparity-defined contour layers. Some further cases involving smooth surface completion between three or more layers are shown in Figure S10.

**Figure 3.**
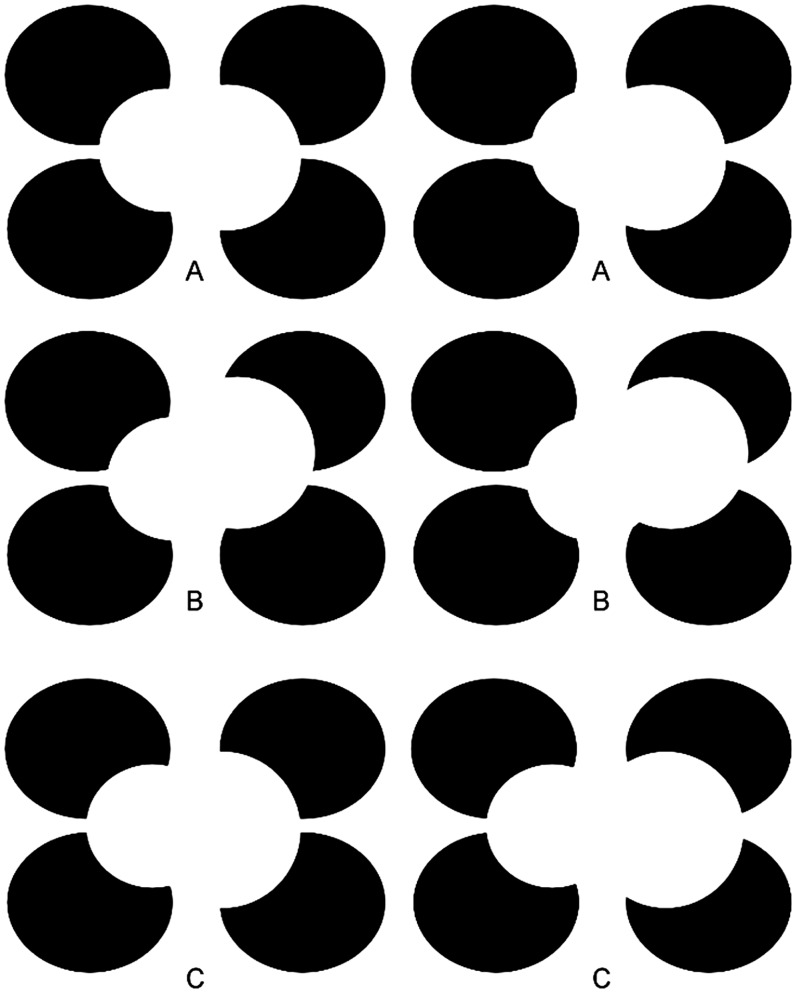
Modal completion examples of 3D surface propagation among boundaries at different disparity-defined depths. All figures are constructed using only two white disks, although perceived volumes are closed differentiable surfaces where image contours are taken to arise from rim rather than edges in the world. Cross-fusion of (A) appears to be a volumetric “thimble,” where (B) looks like a floating peanut shell; (C) also looks like a peanut shell, but one side appears to lie behind and the other in front of the depth layer associated with the black disks. For an uncrossed disparity version of this figure, see Figure S3.

Modal completion examples of volume completion using crossed disparities are shown in Figure 3. (For an uncrossed disparity version, see Figure S3.) In Figure 3(A), the emergent volume looks like a 3D “thimble” protruding upward and to the left, but the image pair was in fact constructed using just two overlapping white disks placed at different disparity-defined depths. The good contour continuation (Tse, 1998; Tse, 1999a, 1999b) between the boundaries of these two disks permits the interpolation of a virtual boundary within which the thimble’s volume can be interpolated between real and illusory contours all taken to arise from surface rim rather than edge. In Figure 3(B), two white disks in a different arrangement give rise to the percept of a 3D closed and differentiable “peanut shell.” And in Figure 3(C), the same shape emerges but one half lies in front of and the other half lies behind the black disks. Under an overlapping disks interpretation, visible contours would arise from edges. But under volumetric interpretations, contours arise from the rim, permitting surfaces to continue both in front of and behind the rim to close into a volume. The completion in front of the rim can be thought of as modal completion and the completion behind the rim can be thought of as self-amodal completion because of self-occlusion. That both processes happen together in the process of volume formation suggests that the two processes may be inseparable. That the interpolated surface can curve from visible or illusory rim segment to rim segment can lead to the paradoxical situation that the front of the interpolated volume can be perceived to lie closer to the viewer than the disparity-defined depth of the rim itself, whereas the presumed back of the object can seem to lie further away than the real or illusory contour inferred to arise from the rim.

**Figure 4.**
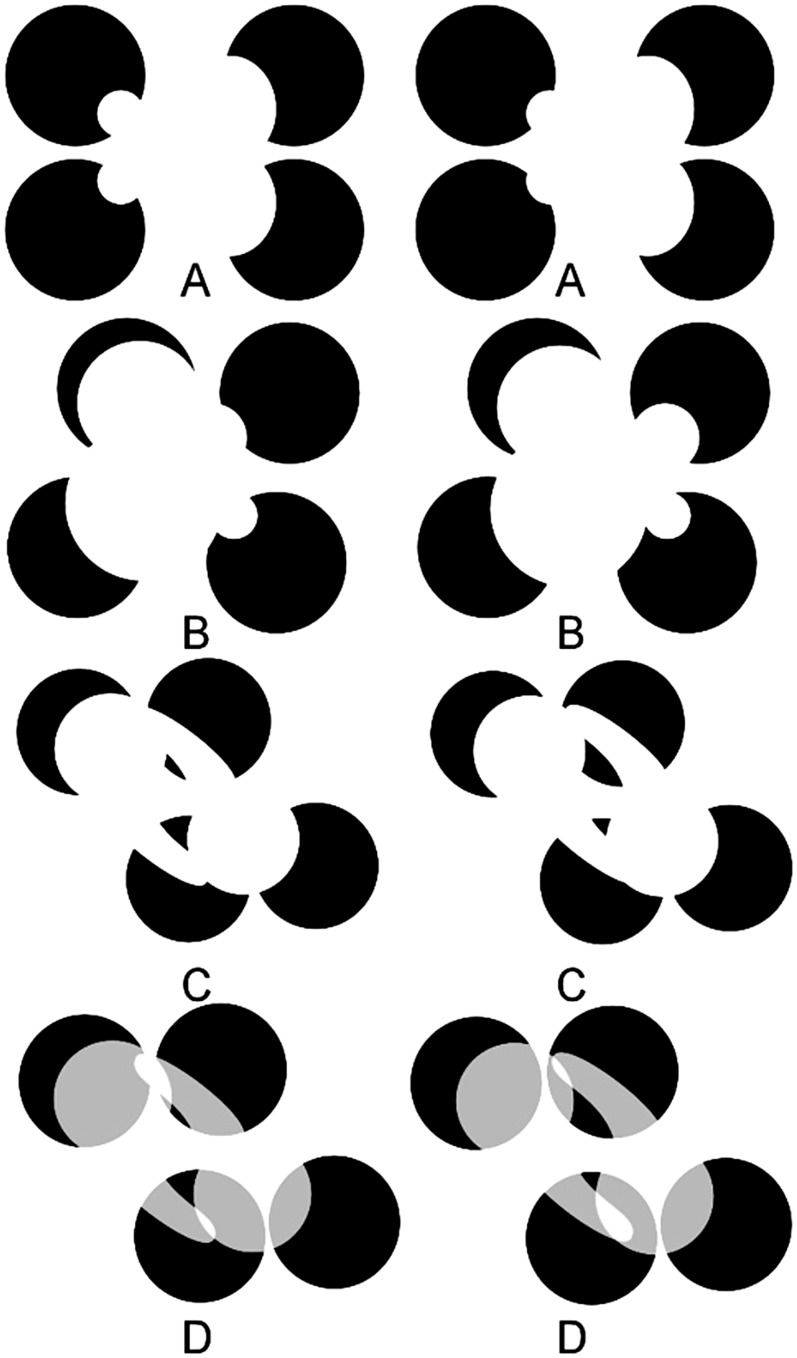
(A) to (C) involve cross-disparity cases where 3D interpolated volumes appear to occlude one another. The occlusion relationships are ambiguous, and top-down visual processing can result in different volumetric shapes and occlusion relationships among the interpolated volumes perceived. (D) is identical to (C) except that the occluding regions are now consistent with the presence of a transparent layer, allowing the formation of a translucent volume. Other examples of such stereo-defined translucent interpolated volumes can be seen in Figure S10. Note that in all cases, only two depth planes are defined by disparities of the occluding figural subportions that occlude the “pacmen,” but the visual system fills in interpolated closed 3D differentiable surfaces (volumes) between these two depth planes. For an uncrossed disparity version of this figure, see Figure S4.

The cases shown in Figure 4(A) to (C) show crossed disparity cases of illusory volumes that modally and amodally complete over one another. For an uncrossed disparity version of this figure, see Figure S4. Figure 4(A) and (B) were created by placing four white disks or ellipses on top of black disks on a white background. But because of good contour continuation and the absence of “blocking” contours between the visible contours placed at different disparity-defined depths, the visual system interpolates gourd-like volumes between the visual surface fragments. The good contour continuity relationships allow more than one interpretation, and with top-down control, one can choose to see more than one set of 3D shapes occluding one another. But in all 3D interpretations, the volumes appear to conform to one another (Albert & Tse, 2000). Figure 4(D) offers a version of Figure 4(C) with added cues to transparency, which gives rise to the percept of two illusory translucent volumes occluding one another upon binocular fusion. Further examples of illusory translucent volumes are shown in Figure S10.

## Dynamic Modally Completing Volumes

When contours are taken to arise from the differentiable rim rather than nondifferentiable edges, interpolated surfaces can continue both in front of and behind the rim to close into a closed surface or volume. If static inducers are placed into relative motion, as in Movies M1 and M2 (fast, crossed disparity), M3 and M4 (slow, crossed disparity), and M5 and M6 (slow, uncrossed disparity), the interpolated volumes can appear to deform in 3D to accommodate inducer motions. The issue of dynamic volume completion and deformation is taken up in greater detail in the companion paper (Tse, 2017) in this special issue of *i-Perception* on amodal completion.

## Clay Modeling of 3D Percepts

**Figure 5.**
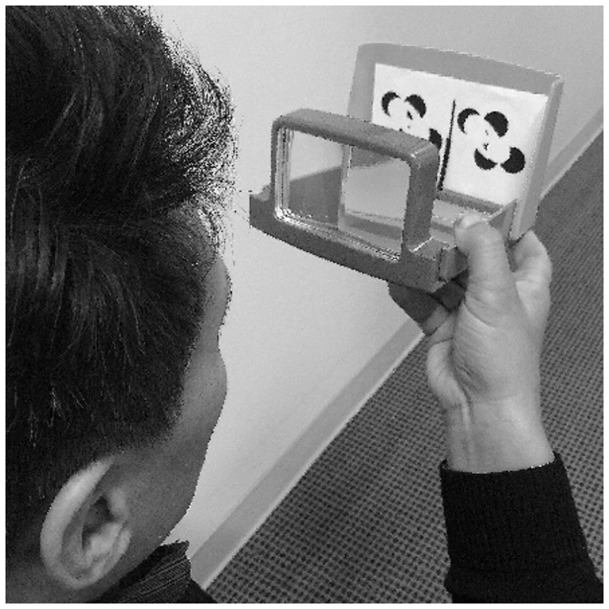
The stereogram viewer used for clay modeling.

**Figure 6.**
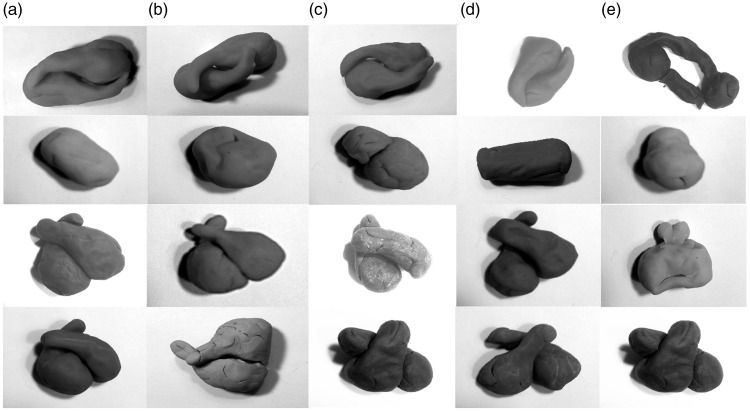
Columns refer to five individual naive subjects who modeled their percepts in play-dough. Row 1: Figure 4(C); Row 2: Figure 3(A); Row 3: Figure 4(A); Row 4: Figure 4(B).

It is not clear what kind of psychophysical data the field of vision research would have demanded from Gaetano Kanizsa concerning his famous Kanizsa triangle demonstration. If his goal was to prove that people see an illusory triangle complete with illusory contours, then in one sense, the demo itself provides compelling data because most people see a triangle where in fact there is none. Here, I faced a similar quandary, in that those who see volumes in the cases presented here find these demonstrations compelling. But, at least when using the stereogram viewer shown in Figure 5 (Loreo Z-GL2001-VAA Deluxe 3D Stereo Print Viewer), not all subjects were able to fuse the two images into a binocular percept. The 3D shapes shown in Figure 1 were presented to naive subjects using the stereogram viewer. All subjects were run under a Dartmouth internal review board–approved protocol for human visual psychophysics, and gave consent before being asked to participate. Of nine subjects who viewed the images in Figure 1, five were able to perceive a curved triangle or cone. The others were either not able to fuse the images appropriately or reported seeing “triangles” without spontaneously reporting seeing curved triangles in 3D. This is consistent with data that nearly a third of subjects have stereo vision anomalies in some domain of disparities or outright stereo-blindness (Richards, 1970, 1971; Van Ee, 2003). This step acted as a filter in the experiment of interest to only examine subjects capable of stereopsis. These five subjects, labeled A to E in Figure 6, were from a wide range of ages (A–E, ages 49, 45, 16, 15, and 10 years old, respectively). They were then given various exemplars from Figures 3(A) and 4(A) to (C), and were given, for each new figure, a three-ounce container of play-dough and were told to, as precisely as they could, model the 3D shape of the occluding white shapes that were blocking the occluded black disks, using all of the play-dough given. No further instructions or corrections or feedback were given.

As illustrated in Figure 6, the five naive modelers generally completed figures that involved smooth closed surfaces that connected the volume across depths. At the bottom of Figure 6, there are letters corresponding to each individual. The rows correspond to the four models made by each of the five modelers. While the models are largely similar, there are differences. For example, in Row 3, corresponding to Figure 4(A), Subject E reported seeing a different 3D shape than the other subjects, and in Row 4, corresponding to Figure 4(B), Subjects A and B reported seeing the two figures overlap one way, whereas the other three subjects reported seeing them overlap the other way in 3D. These differences likely arise from the fact that there are several possible 3D volume percepts in each case that are all consistent with the rim relationships implied by image contours, and subjects simply reported different interpretations.

## Discussion

What could account for 3D surface interpolation between visible contours at two discrete depths? That the surfaces are smooth implies that “countless” depths (with implied disparities) are represented on the basis of just two discrete depths available in the image pair. Such interpolation of smooth surfaces into regions that themselves lack disparity information must derive from the visible contour fragments and their depth information because this is the only such information available in the stimulus. But the visible contour fragments and their respective depths are consistent with many possible 3D interpretations. So there must be internal constructive processes that give rise to the particular 3D shape solutions that are perceived. But what exactly is the nature of these internal processes?

Several ideas have been put forth regarding surface interpolation processes in both the psychological and computer vision fields. Some surface completion algorithms (Sarti, Malladi, & Sethian, 2000) have been able to account for the flat surface perceived in the Kanizsa triangle by viewing the problem as one of minimizing surface curvature of a Riemannian manifold whose metric properties are constrained to meet conditions imposed by image contours. The first step in almost all such algorithms is to detect image contours. This is followed by a step of surface evolution that is attracted to edges, and which also completes missing edges and surfaces among and between visible edges following a surface smoothness constraint that follows from the minimization of Riemannian curvature, as would occur for a soap bubble suspended between wires at the locations and depths of the image contours.

But in the cases considered here (for further examples see Figures S1–S12), the surfaces interpolated by the visual system behave very differently than soap bubbles hanging among wires defined by visible contours. Unlike soap bubbles, surfaces in Figures 2 to 4 appear to pass through visible contours along the line of sight tangentially as would occur when looking at a smooth closed surface (e.g., a potato), rather than orthogonally to it, as in the traditional Kanizsa triangle case. That is, in the cases demonstrated here, contours are not taken to arise from edges, where surfaces just end. Instead they are taken to arise from the rim, where the line of sight tangentially grazes a smooth surface that continues smoothly away from the rim, in both forward (toward the viewer) and backward (away from the viewer) directions. Volume formation may proceed because, at the rim, surfaces continue into self-occluded space and can wrap around and meet other such rim surfaces, in a form of self-modal and self-amodal completion.

Rather than propagate surfaces toward visible contours, other algorithms propagate curved surfaces inward from visible contours. At least one such contour curvature propagation algorithm (Tse, 2002) depends on the availability of contour curvature discontinuities that could have arisen from a planar cut of a 3D surface, which can then reveal information about the 3D cross section of a volume. But the disks used in the present demos do not carry any such contour curvature discontinuity information in their contours, so cannot be used to infer the shape of a cross section of a volume according to this algorithm (unless a disk is taken to arise from a frontoparallel circular cross section).

An alternative idea is the idea of an “attentional shroud” (Cavanagh, Labianca and Thornton, 2001; Fazl, Grossberg, & Mingolla, 2009; Moore & Fulton, 2005; Tyler & Kontsevich, 1995) that places a mesh among visible contours, and which can have a certain rigidity among nodes of the mesh, limiting it from collapsing into a soap bubble solution. But rigidity of a default surface mesh cannot easily account for the fact that sometimes interpolated surfaces pass through visible contours that lie tangent to the line of sight (i.e., at the rim), as in Figures 3 and 4, and other times are interpolated to lie orthogonal to the line of sight as in the examples shown in Figure 1 (i.e., at a surfaces edge). Future theoretical work will have to explain why 3D open surfaces are interpolated for the examples shown in Figure 1, but why closed surfaces or volumes are interpolated for the other figures here.

While speculative, the idea of an attentional shroud or 3D encompassing surface or manifold could in principle be realized in something like the “grid cells” (Moser et al., 2014) known to specify a coordinate system for a 3D layout. However, to date no one has found or even proposed analogous “mesh cells” for 3D objects or surface representations of objects. If such cells exist, which would lay down a 3D mesh within visible contours of objects, in a manner analogous to the laying down of a grid by grid cells within visible borders, a possible place to look for them might be among the recently described (Yau, Pasupathy, Brincat, & Connor, 2013) 3D surface curvature cells in visual area V4. At this point, however, the existence of such cells is purely speculative.

Certain models have made explicit that surfaces can be completed from visible contour fragments within and between depths. Notable among these is the Boundary Contour System/Feature Contour System (BCS/FCS) model of Grossberg and Mingolla (1985) and the later elaboration of that theory called “FACADE theory” (Grossberg, 1994). These models posit “bipole cells” that complete contours that are adequately coaligned based on good contour continuation in the image. If that level of completion fails, then the second stage of surface “diffusion” away from the completed contour does not take place. And even if it begins, diffusion can get blocked by a visible boundary (Mumford & Shah, 1989; Neumann, Pessoa, & Mingolla, 1998; Perona & Malik, 1990; Proesmans & Van Gool, 1999). Problems arise for such models in that contour continuity can occur in the image that arises when two occluded volumes are in fact not connected in the world, contour continuity can occur but surface completion fails for other reasons, and a volume can complete even when there are no coaligning contours in the image at all (Tse, 1998; Tse, 1999a, 1999b). Thus, contour continuity in the image is neither necessary nor sufficient for amodal or modal completion. More recently, some authors (e.g., Kogo, Strecha, Van Gool, & Wagemans, 2010) have emphasized that depth ordering is the primary problem that must be solved, followed by surface completion within the contours at a given depth. But this approach still cannot account for surfaces that complete smoothly behind, between, and even in front of the depth planes explicitly given by image contour disparity cues, as occurs in, say, the witch’s hat example, in part because such models treat contours in the image as having arisen from edges in the world, rather than from the rim. Whereas most modeling to date has focused on cases where contours arise from surface edges, future modeling should attempt to construct surface meshes interpolated away from contours that are taken to arise from rim.

Whether any local portion of image contour is interpreted to arise from a surface edge or surface rim cannot be decided based only on local cues. The rim interpretation, where the line of sight is taken to tangentially graze a differentiable surface, requires that a distal portion of rim exists to which the surface can link as a smooth manifold that can bend as much as 180° in space. But if one counts the self-occluded regions of the closed surfaces perceived in many cases considered here, the surface inferred to pass through the rim must bend through 360° in order to close in on itself from the far, self-occluded side.

**Figure 7.**
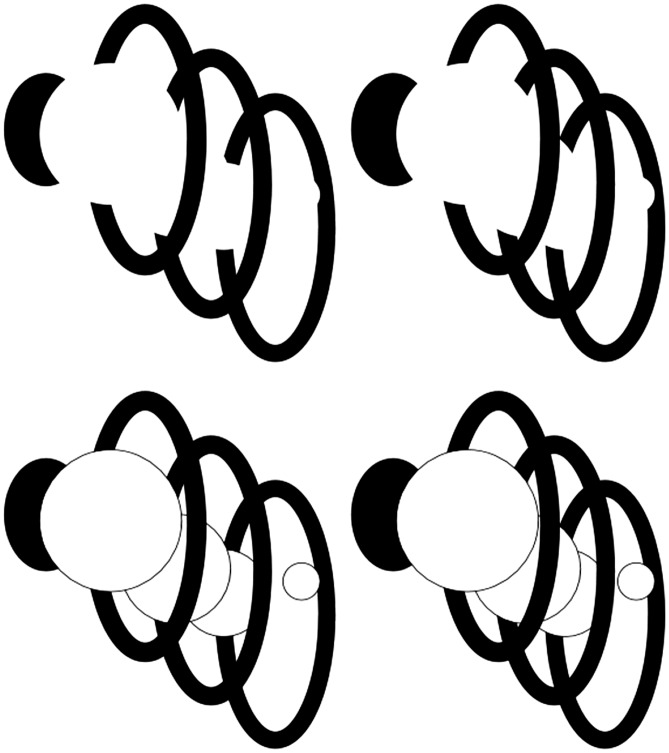
Upon cross-fusion, the percept of a red-hot chili pepper passing through rings, as in the top row, is eliminated if explicit contours are added to the circles, as in the bottom row. For an uncrossed-disparity version of this figure, see Figure S5.

One might think that any visible contour could block surface propagation. But only contours that are owned by an object or layer can block propagation. Contours that belong to an occluding Object X do not block surface completion from one contour segment arising from inferred rim of Object Y to another such segment at a different depth plane. For example, in Figure 7, made using crossed disparities (for an uncrossed disparity version of this figure, see Figure S5), the contours arising from the black rings do not block a volume from forming in the 3D shape of a red-hot chili pepper that passes through the rings, even though adding contours to the circles, as in the bottom row of Figure 7, leads to a very different percept.

In conclusion, the present demonstrations extend and go beyond the insights about visual processing raised by Gaetano Kanizsa’s (1955) triangle figure or the open curved surfaces introduced by Carman and Welch (1992). Unlike those classes of examples, the present class of examples makes plain the extent to which surface interpolation can involve a 3D volume completion process, not limited to the flat surfaces found in the Kanizsa triangle. In the present class of demonstrations, smooth surfaces are interpolated to span two or more discrete disparity-defined depths. That two or more discrete contour fragments at discrete disparities can give rise to an analog representation of a unified and closed 3D surface is remarkable and is a testament to the daily creativity of the constructive processes that underlie everyday 3D vision (see Koenderink, 2015 for more on this theme). It must be granted, however, that no existing theoretical or computer models of visual processing can fully account for the 3D surfaces and volumes perceived in the present demonstrations. It can only be hoped that future modeling and neurophysiological research will be able to explain why these 3D surfaces are perceived rather than the many others that are also consistent with the present image cues.

## Supplementary Material

Supplementary material

Supplementary material

Supplementary material

Supplementary material

Supplementary material

Supplementary material

Supplementary material

## Note

1 Some observers report seeing the lower left corner of the interpolated cone-like object in Figure 1(A) as flattened, as if one had opened a conical paper coffee drip-filter while pinching down the conical end. This suggests that the surface curvature information from the right-hand edge fails to propagate into this region, probably because there is no disparity among local cues to shape in the two eyes’ images.
