# Supplementary figures and images for: Volume Completion Between Contour Fragments at Discrete Depths
Source: Iperception. 2017 Dec 21;8(6):2041669517747001. doi: 10.1177/2041669517747001 (PMC5753925; doi:10.1177/2041669517747001)

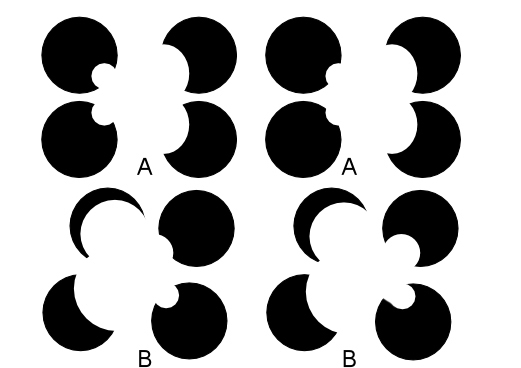

Supplement: Supplementary material [file Supplementary_Movie1.gif]

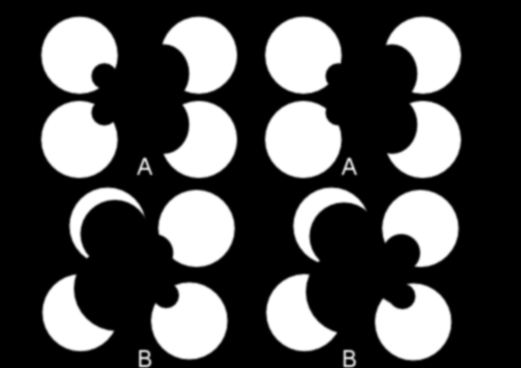

Supplement: Supplementary material [file Supplementary_Movie2.gif]

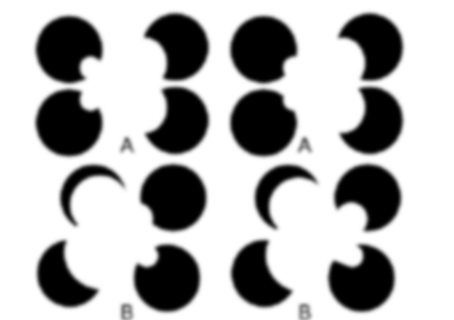

Supplement: Supplementary material [file Supplementary_Movie3.gif]

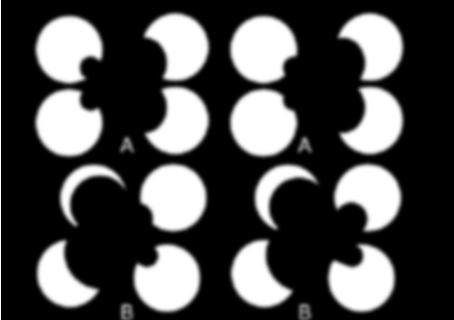

Supplement: Supplementary material [file Supplementary_Movie4.gif]

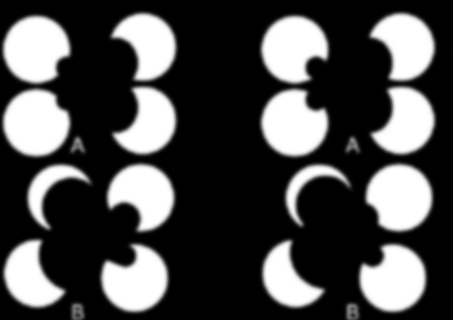

Supplement: Supplementary material [file Supplementary_Movie6.gif]

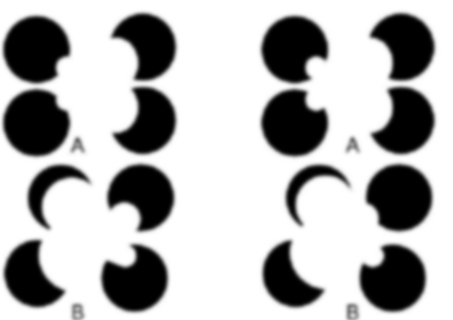

Supplement: Supplementary material [file Supplementary_Movie5.gif]
