# Supplementary material for: Volume Completion Between Contour Fragments at Discrete Depths
Source: Iperception. 2017 Dec 21;8(6):2041669517747001. doi: 10.1177/2041669517747001 (PMC5753925; doi:10.1177/2041669517747001)
Supplement: Supplementary material [file TseVolumesPerceptionFinalUpdated2.pdf]

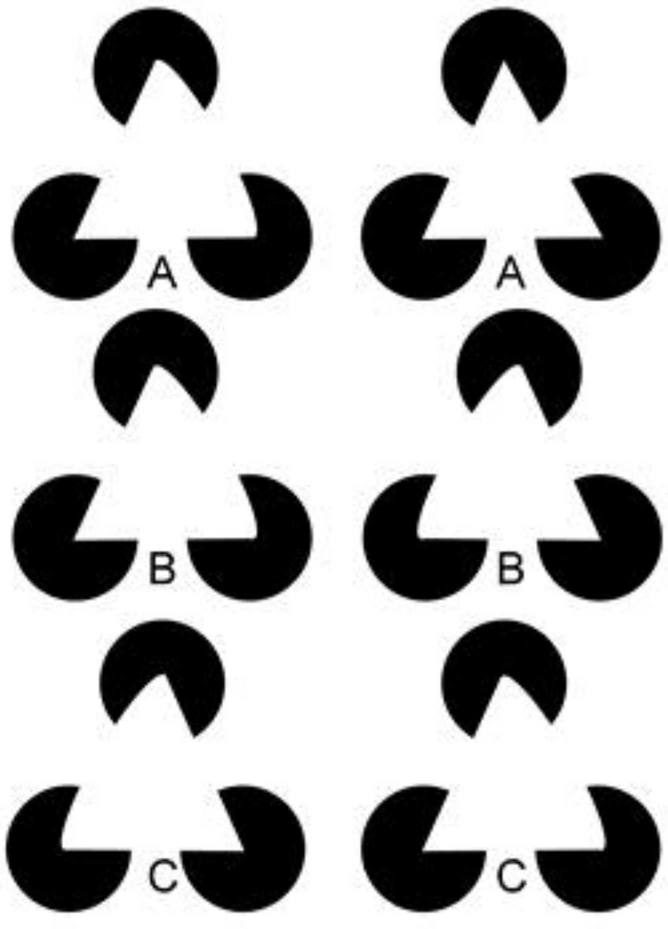

Figure S1. An uncrossed-disparity version of Figure 1.

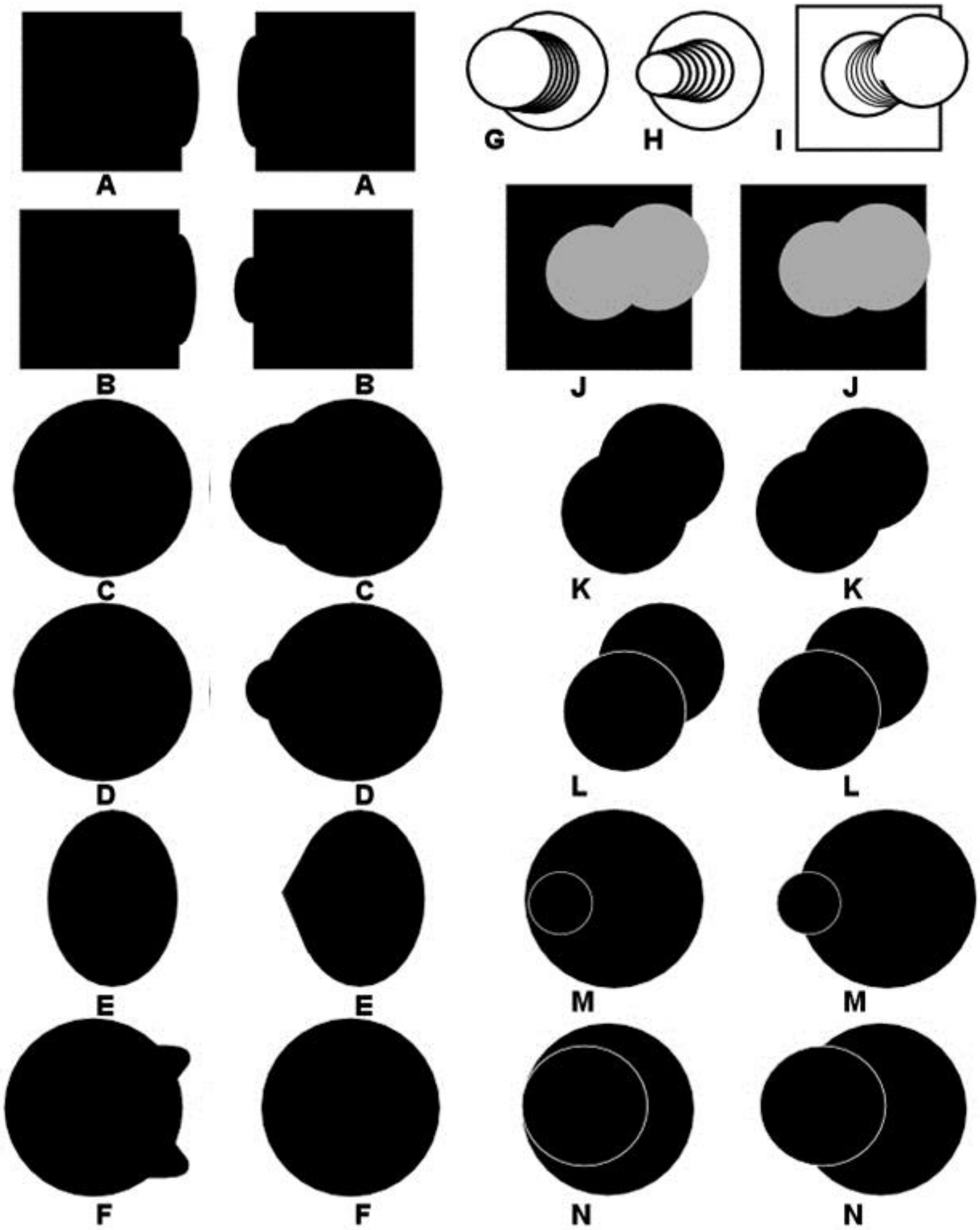

Figure S2. An uncrossed-disparity version of Figure 2. When viewed with uncrossed disparities, these look like smooth volumes or closed differentiable surfaces, but when viewed with crossed disparities, these generally fail to form volumes, but instead look like unclosed surfaces occluding other unclosed surfaces, and, in the modal cases, apparently through oval windows.

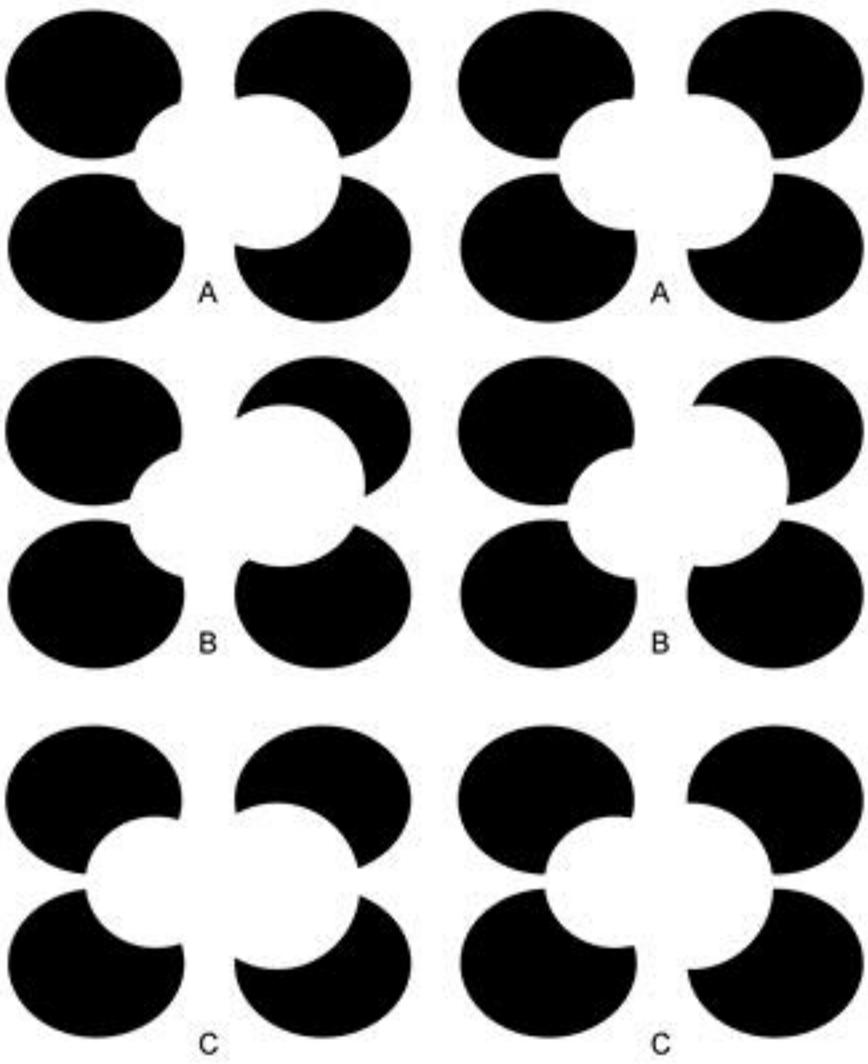

Figure S3. An uncrossed-disparity version of Figure 3.

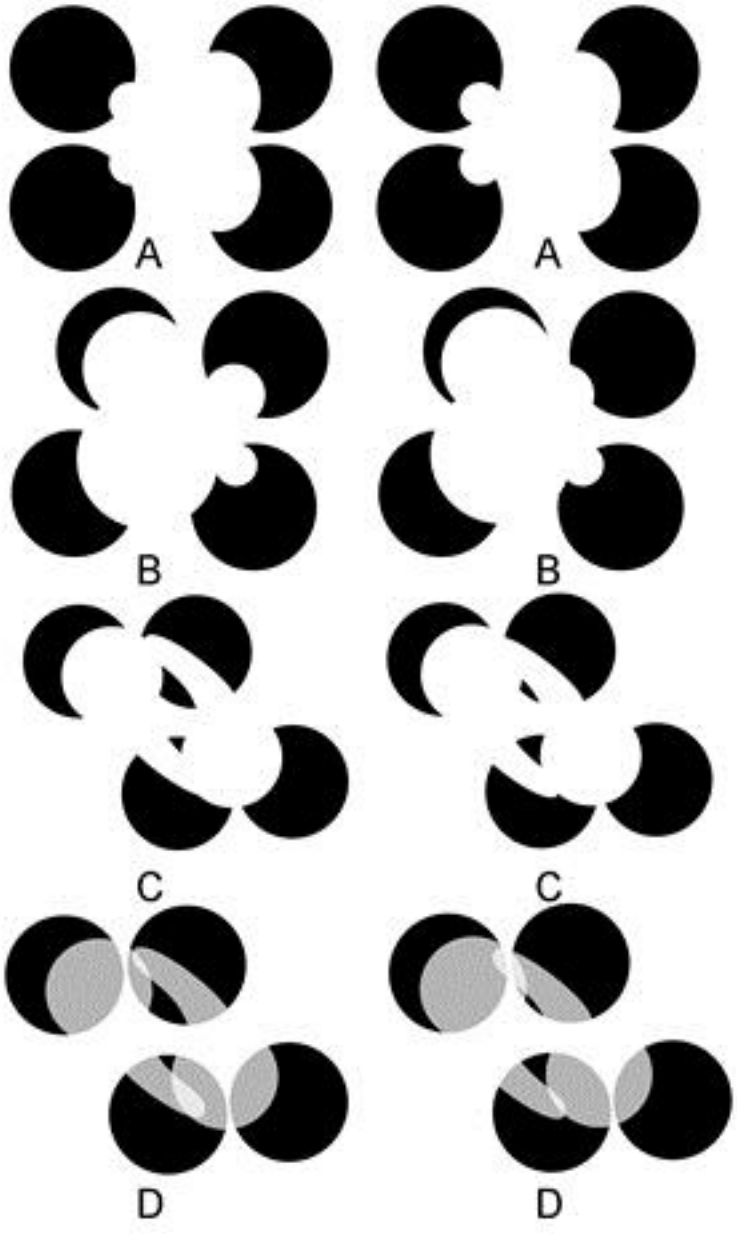

Figure S4. An uncrossed-disparity version of Figure 4.

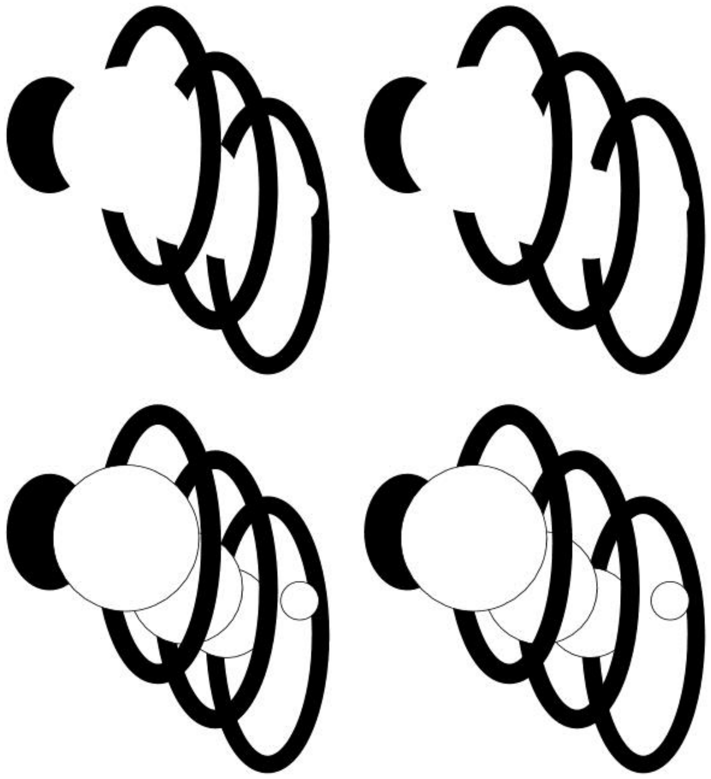

Figure S5. An uncrossed-disparity version of Figure 7.

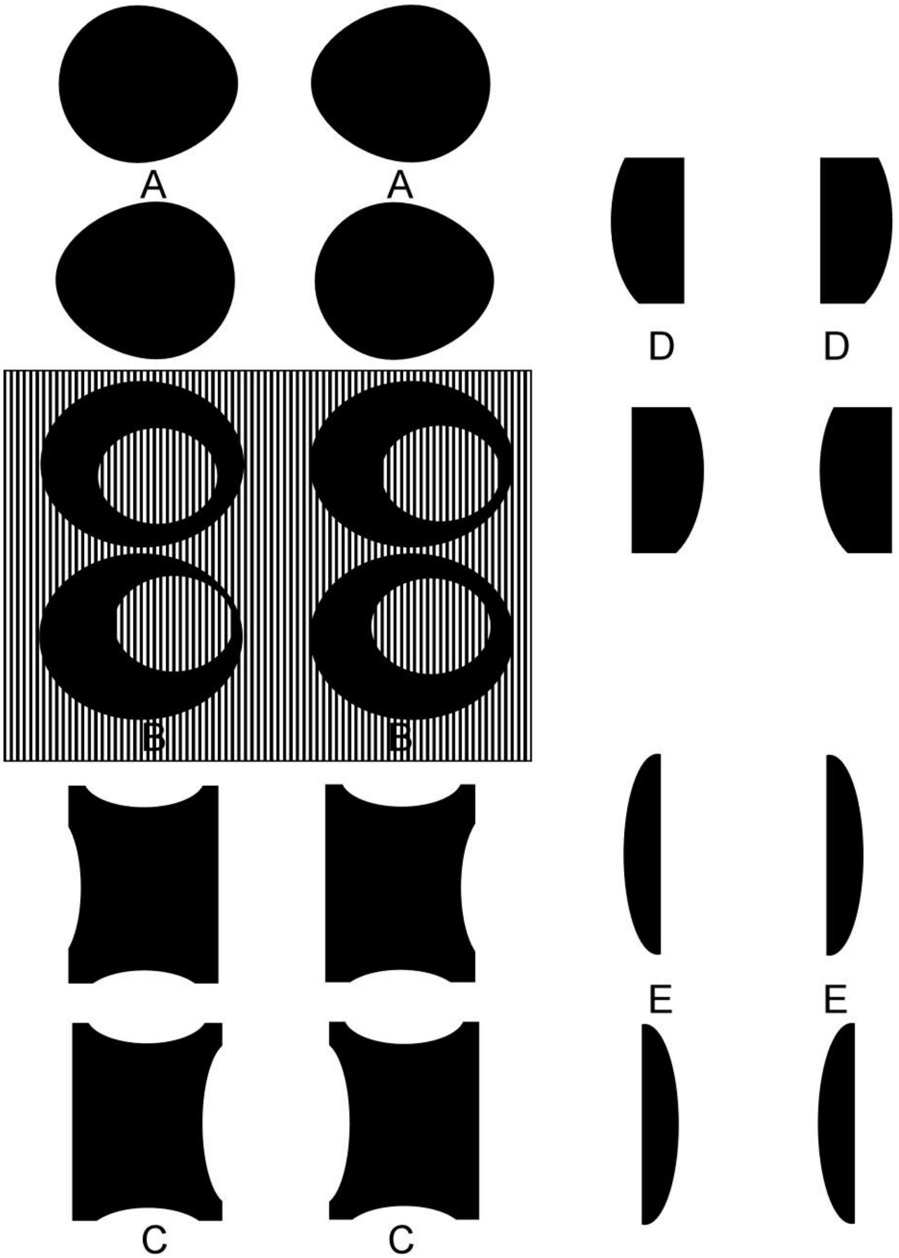

Figure S6. Further examples of curved surfaces generated using crossed-disparity stereopsis. See Figure S7 for an uncrossed disparity version of this figure. The cases A, B, D and E are not volumetric and also do not involve unmatched contour points in the two images. Case B is interesting because this completes into a ‘lampshade’ or a ‘pipe’ depending on the disparity differences of the two visible circles, suggesting that a sheath is interpolated to connect each point on a visible circle to a corresponding point on the other circle. Case C appears to complete into a volume in the shape of a Japanese bridge viewed either from below or above. This was drawn simply by placing white ellipses in front of a black rectangle. But the resulting Japanese bridge percept is radically different from ellipses in front of a rectangle.

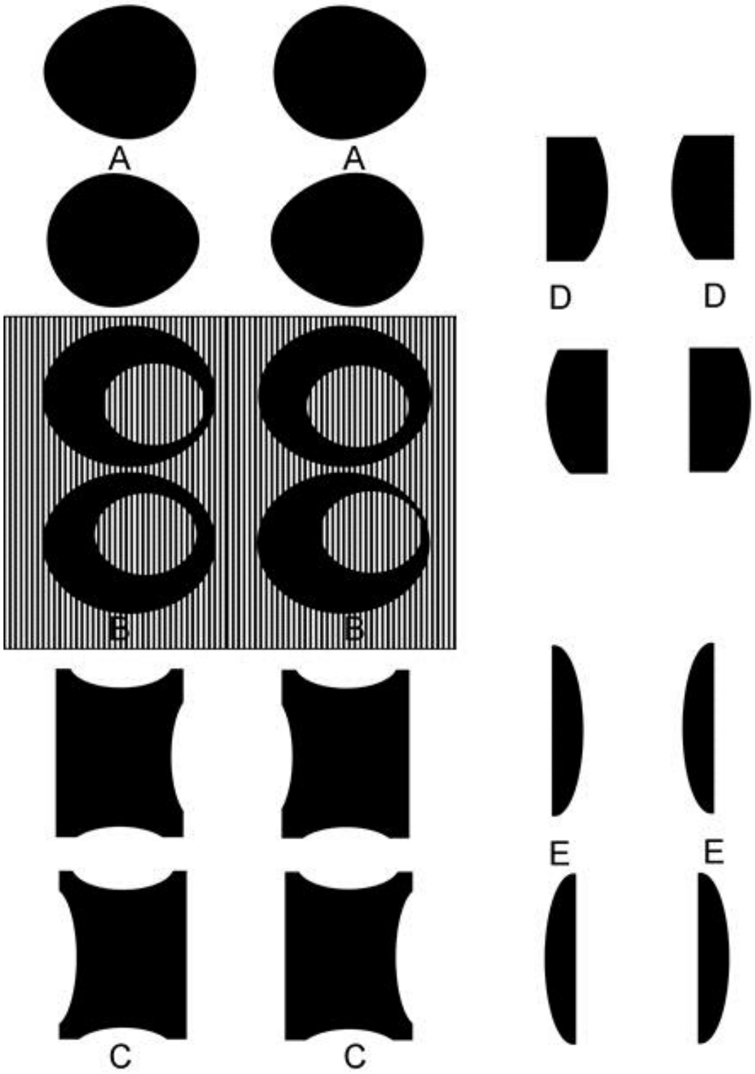

Figure S7. An uncrossed-disparity version of Figure S6.

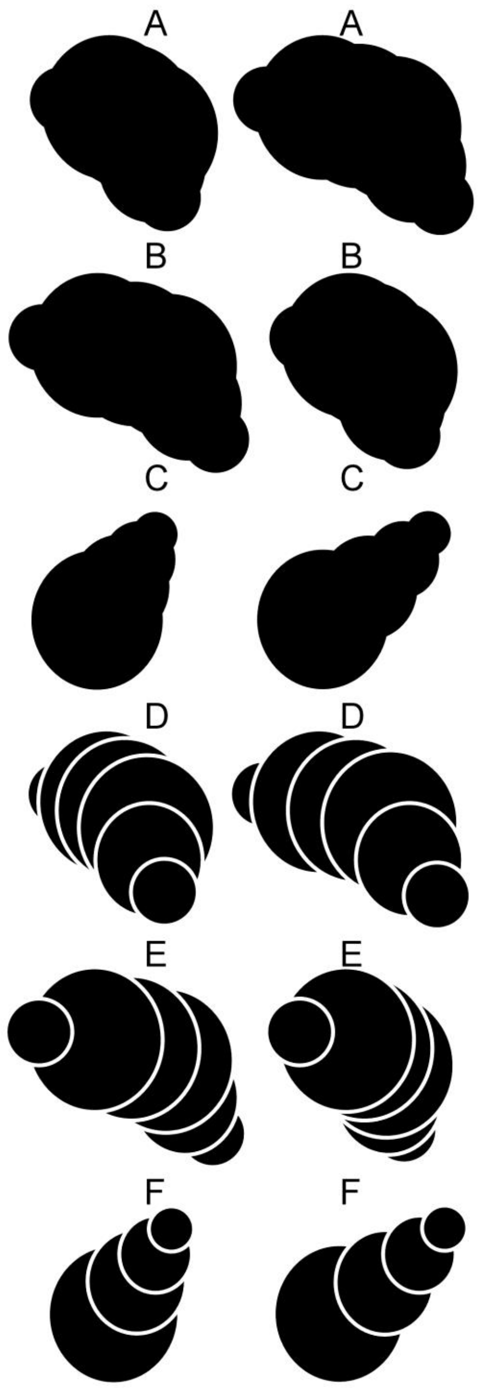

Figure S8. Whereas the cross-disparity examples in the main text involve volume completion across contours placed at two discrete depths, the examples in the top three rows involve cases with multiple discrete depths. When the contours are made explicit, as in the lower three rows, no surface interpolation between layers can take place, and the sole binocular solution is a stack of discretely placed disks. In this explicit contours case the contours are taken to arise from edge in the world rather than rim, whereas without the explicit contours, the ‘rim’ solution dominates the ‘edge’ solution. For an uncrossed-disparity version of this figure, see Figure S9.

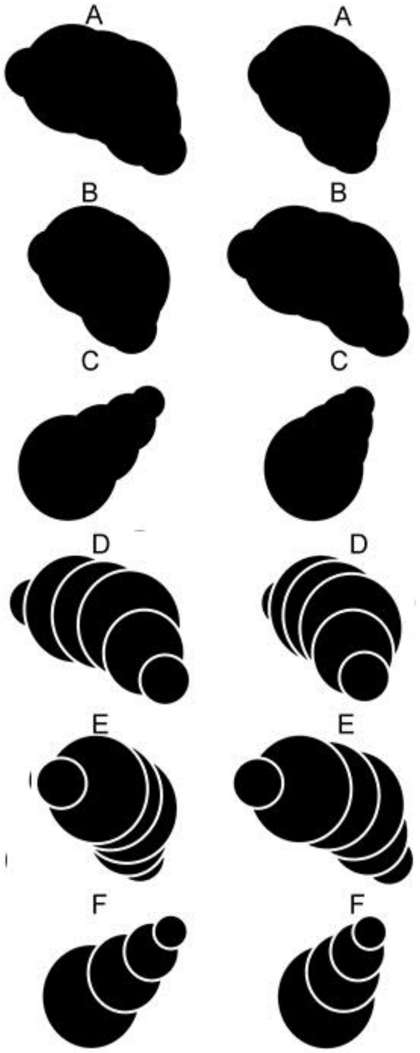

Figure S9. An uncrossed-disparity version of Figure S8.

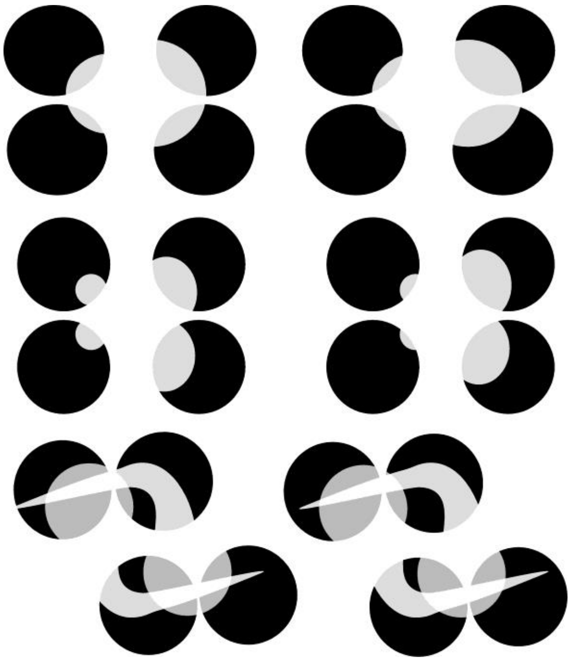

Figure S10. Further examples of interpolated translucent volumes using crossed-disparities. For an uncrossed-disparity version, see Figure S11. The upper case is made to look like a pill capsule pointing upward and to the right, the middle row like ‘chicken legs’ extending upward to the right, and the bottom case like two overlapping translucent herons’ heads and necks.

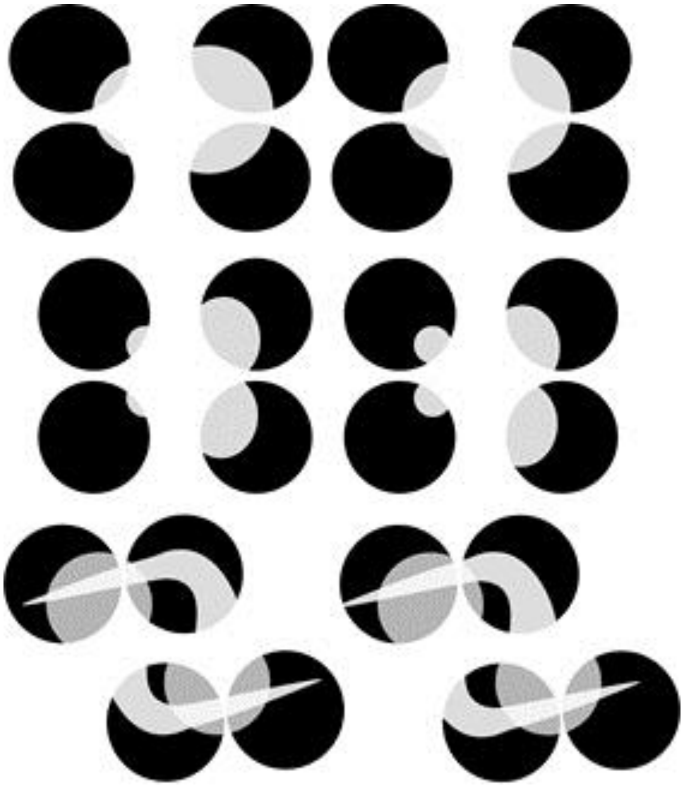

Figure S11. An uncrossed-disparity version of Figure S10.

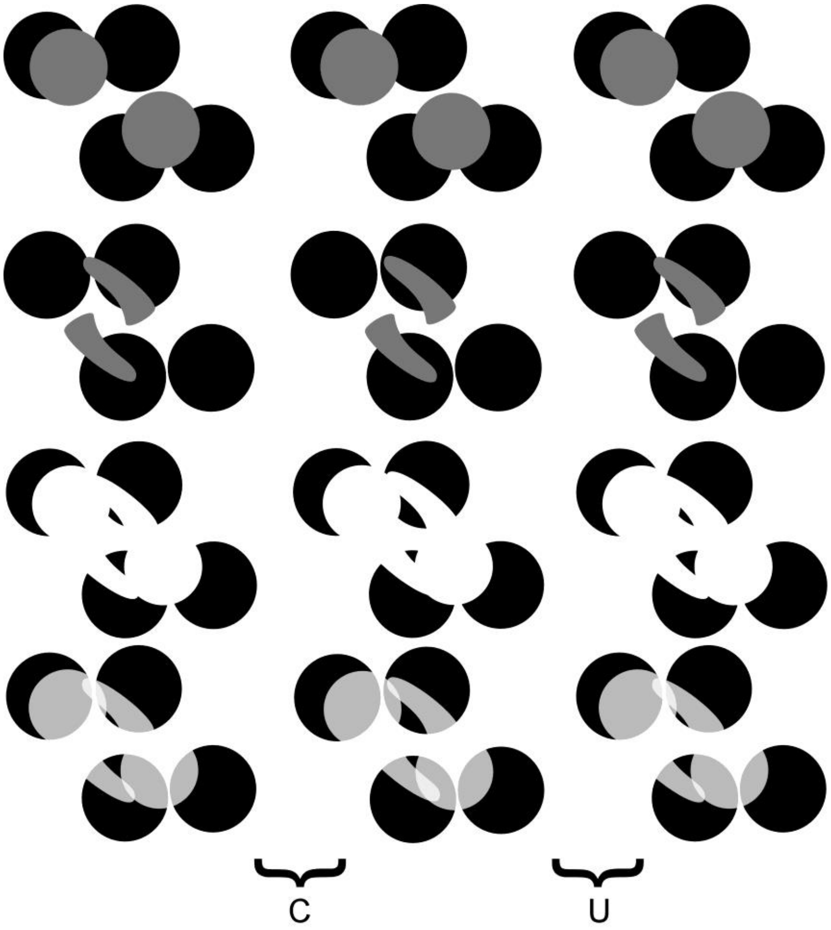

Figure S12. Crossed and uncrossed disparities are shown side by side. The top rows depict the elements used to create the figures, each placed at a discrete disparity.
